# Demanding solidarity, not salvation: sex work and global health

**DOI:** 10.1136/bmjgh-2025-022050

**Published:** 2026-05-19

**Authors:** Marlise Linda Richter, M H Ditmore, Jantina de Vries

**Affiliations:** 1 The EthicsLab, University of Cape Town Department of Medicine, Observatory, South Africa; 2 African Centre for Migration & Society, University of the Witwatersrand Johannesburg Faculty of Humanities, Johannesburg, South Africa; 3 Principal of Clear Eyed Consulting, New York, New York, USA

**Keywords:** COVID-19, Global Health, Social Discrimination, Universal Health Care, Delivery of Health Care

## Abstract

There is increasing attention paid to solidarity in global health, but its substance and definitions remain contested. We explore the tensions between global health institutions’ historic approaches to sex work, their commitment to health and human rights and how these are connected to or disconnected from solidarity. We foreground the protracted and incomplete evolution from international health approaches to sex workers as spreaders of pathogens that should be punished, to sex work health programmes that are situated within human rights principles. Thus, substantial resources and material changes to laws, policies and programmes are required to action claims of ‘standing in solidarity’ with sex workers. We argue that the drastic cuts to global health funding initiated by the Trump Administration in January 2025 require careful consideration of what ‘solidarity’ with the most marginalised entails and bold action.

**Summary box**

- Solidarity in Global Health requires critical analysis and a consideration of diverse approaches and world views.
- Employing solidarity as a lens through which to view global health dynamics helps identify the greatest vulnerabilities and dangers to collective health and to examine underlying power structures and resources.
- Historically, international health programmes have approached sex workers as ‘vectors of disease’ rather than as holders of rights.
- Solidarity with sex workers requires a principled commitment to speaking out against ideological-based funding restrictions and the far-reaching impact of US global health funding cuts.

## Introduction: solidarity and its invocations

**Figure 1:**
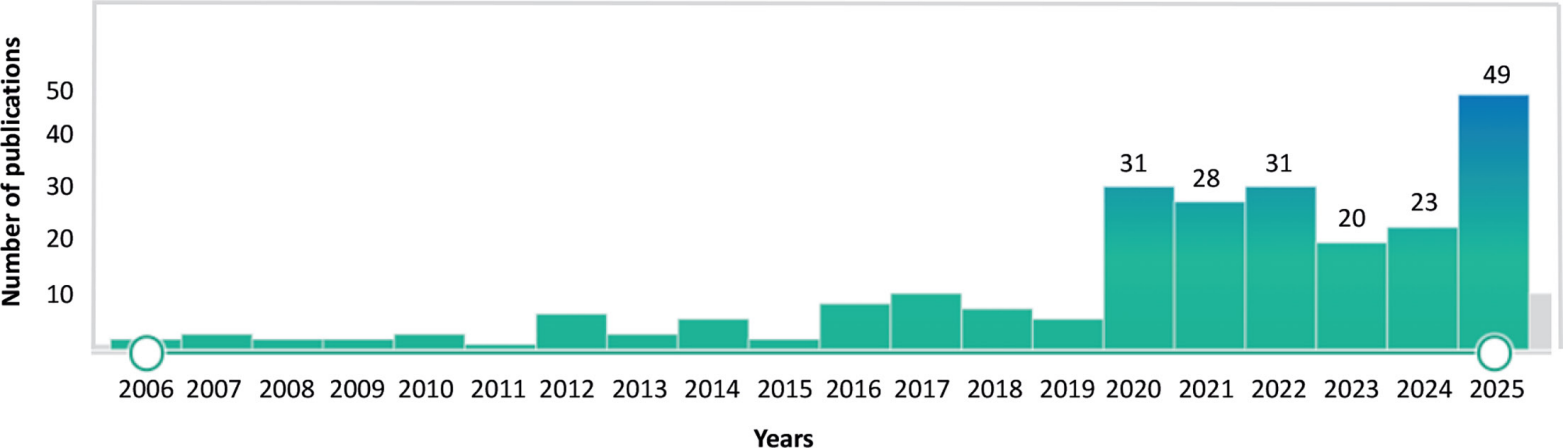
PubMed search of English language articles with the terms ‘solidarity’ (in the title or abstract) and ‘global health’ (in the title or abstract) from 1 January 2006–31 December 2025, n=233 (Figure generated by PubMed and modified to include number of articles published during the COVID-19 pandemic and directly thereafter).

 There is growing interest in the role of solidarity in global health. The COVID-19 pandemic in particular catalysed keen academic and programmatic interest in notions of solidarity, global interconnectedness and health. For example, a PubMed search for “solidarity” and “global health” in the title or abstract in English language articles shows that close to 80% of the 233 articles published (182/233) from 2006 to 2025 were written during or after the COVID-19 pandemic (see figure 1).

Solidarity was frequently invoked during the COVID-19 pandemic—particularly during the early stages of the pandemic and decision on global distribution of COVID-19 vaccines. For some, the global response to COVID-19 constituted a ‘catastrophic moral failure’^1^; wealthy countries in the global north bought sufficient COVID-19 vaccines to vaccinate their populations many times over while global south countries scrounged for access to a fraction of global supplies.^2^ The early WHO slogan ‘We are in this together’^3^—oft repeated by politicians^4^—rang increasingly hollow.

A recent modelling study quantified this deficit of solidarity in stark terms: of the 20 lower- and middle-income countries included in the study, up to half of deaths due to COVID-19 could have been averted if vaccines were more equitably distributed globally.^5^ Appealing to solidarity may have created public reassurances of global commitments to unity and care but left the structural determinants of ill-health and unequal geopolitical power structures untouched, with lethal consequences.

Yet this experience is neither unique nor surprising. In fact, the failure to stand in solidarity with others facing dire health threats globally has played out many times in history and particularly for sex workers. Throughout the HIV epidemic, sex workers and their allies have called for solidarity and the recognition of the interconnection of the human rights and health of all. These calls have often been ignored.

Below we explore the tensions between global health institutions’ approaches to sex work and their commitment to the principles of health and human rights and how these are connected to or disconnected from solidarity. This focus flows from our interest in the protracted and incomplete evolution from international health approaches to sex workers as spreaders of pathogens that deserve punishment to sex work health programmes situated within human rights principles and thus the significant material changes and resources required to action claims of ‘standing in solidarity’ with sex workers. We argue that the recent drastic cuts to global health funding by the Trump Administration require careful consideration of what ‘solidarity’ with the most marginalised entails and bold action.

### Solidarity definitions

Many have an intuitive appreciation of what solidarity entails and may regard it uncritically as a ‘beautiful notion, practised voluntarily by generous and kindhearted persons’,^6^ but formal definitions are contested.^7^,^9^ More discursive and empirical work is required to illuminate and grapple with the complexities of solidarity in various contexts. Here we employ two definitions coined by Prainsack and Buyx: ‘solidarity signifies shared practices reflecting a collective commitment to carry ‘costs’ (financial, social, emotional or otherwise) to assist others’,^10^ as it makes explicit that deliberate action and an investment of resources are necessary to make a claim of solidarity towards others. The authors also emphasise the key element of a recognition of similarity: ‘as enacted commitments to accept costs to assist others with whom a person or persons recognise similarity in a relevant respect’.^9^

### Initial health sector responses to sex work: ‘vectors of disease’

Sex workers (or the dated term ‘prostitutes’) have often been portrayed as ‘vectors of disease’ in public health approaches.^11^ Research and programmatic approaches have traditionally constructed sex workers as ‘a potential hazard for society’^12^ and who are only of interest in relation to mitigating the risk they pose to the spread of HIV/AIDS and other sexually transmitted infections (STIs) to others. Such research and health interventions were not rolled out because sex workers mattered as human beings first and foremost but rather to safeguard the narrow interests and health of non-sex workers. This public health approach was typified by social moralism and the instrumentalisation of sex workers, instead of one that focused on dignity, human rights and a recognition of similarity of the other, following the Prainsack and Buyx^9^ definition.

We offer three examples of sex work, HIV and international global health role players to illustrate the shifting nature of global health approaches to sex work.

#### Forging UNAIDS guidelines on sex work 2007 and 2012

In 2006, UNAIDS/UNFPA held an international consultation on sex work and HIV to draft appropriate guidelines to shape services to sex workers. Sex worker groups worked with UN agencies to include participants from the global south on the agenda to speak about obstacles to successful HIV prevention among sex workers. While at the time, there was increasing evidence of the effectiveness of a harm reduction and rights-based approach to HIV programming with sex workers^13^—which was also advanced during the consultation—the 2007 UNAIDS Guidance Note on HIV and Sex Work^14^ subsequently produced focused on the ideological position of reducing sex work or ‘emphasising alternative livelihoods (but without offering any real-life examples of successful alternative livelihood programmes or discussing the human rights pitfalls of these approaches)’.^15^

An alternative position would have recommended the implementation of proven-effective measures that prevent HIV transmission without normative articulation about the acceptability or otherwise of sex work. The 2007 Guidance Note was widely critiqued by civil society organisations and sex worker groups, who urged its withdrawal.^16 17^ One health and human rights organisation noted:

“The Guidance Note seems to back away from a focus on those groundbreaking programmes of, by and for sex worker organisations, some of which have advanced the human rights of sex workers well beyond their impact on HIV. Instead, the document takes positions that feed into the misguided and dehumanising idea that abolishing sex work is a useful approach to preventing HIV”.^15^

Ultimately the UNAIDS Programme Coordinating Board did not approve the 2007 Guidance Note and in 2009 a new Guidance Note was released, which regrettably still contained some ideological components.^18^

In 2012, UNAIDS signalled a new approach: it issued a new Guidance Note that was firmly situated in the input from the ‘Global Working Group on HIV and Sex Work Policy’ comprised of sex workers and sex work projects.^19^ This Note explicitly supported decriminalisation of sex work and changes to the structural inequalities that marginalise sex workers and cause ill-health, all based on evidence. This partnership-based approach was replicated in subsequent UNAIDS programming, including the launching of the ‘Implementing Comprehensive HIV/STI Programmes with Sex Workers: Practical Approaches from Collaborative Intervention’.^20^ It is premised on respectful, rights-based programming with sex workers and community empowerment, addressing violence against sex workers, community-led services, condom programming, clinical services and capacity building, and that had demonstrated effectiveness.^21^

#### The 2012 International AIDS Conference

In July 2012, the XIX International AIDS Conference—a key forum for sharing the latest HIV/AIDS evidence and characterised by civil society activism—was held in Washington despite well-known US visa barriers to entry for sex workers and people who use drugs (PWUD). US travel restrictions barred sex workers and PWUD from entering the country under provisions entitled ‘Crimes Involving Moral Turpitude’.

In protest, international sex worker groups convened a parallel conference in Kolkata, India called the Sex Worker Freedom Festival, co-sponsored by UNAIDS, UNFPA, the IAS2012 conference committee and others. The Kolkata conference report was entitled ‘Solidarity is not a crime’,^22^ thus invoking the importance of solidarity in advocating for sex worker rights and dignity in all forums, while also underlining how restrictive policies and laws circumscribed sex workers’ ability to participate in forums that would make decisions about their lives. Kolkata was selected because its 65 000-strong organised sex worker movement demonstrated what could be accomplished with solidarity: decreasing violence against sex workers, controlling HIV among vulnerable groups, creating a credit union and more.^23^

If the global health community were in solidarity with sex workers in line with the Prainsack and Buyx definitions above, the US would not have been considered as an official AIDS conference host country as it foreclosed international sex worker participation. In contrast, the Freedom Festival built solidarity among sex workers as the collective name given to delegates—‘Sex workers without borders’—suggests, while it highlighted the global ramifications of exclusion from the host country.

#### Ideology-driven donor agendas: the PEPFAR Anti-Prostitution Pledge and the disastrous consequences of the 2025 US funding cuts

In South Africa (SA) in the mid-1990s, specialised sex worker sexual and reproductive healthcare services were established in inner-city Johannesburg^24^ and, following their impact on sex worker access to healthcare services and reducing the risk of HIV, the model was subsequently replicated at scale. This expansion was made possible by foreign assistance HIV funding through USAID/PEPFAR, the Centres for Disease Control (CDC) and the Global Fund. PEPFAR funds, however, imposed rigorous ideological requirements—popularly called the ‘PEPFAR Anti-Prostitution Pledge’—that were at odds with public health evidence.^25^ ^26^

The United States Leadership Against HIV/AIDS, Tuberculosis, and Malaria Act of 2003 (‘Leadership Act’) provided that:

“No funds … may be used to promote or advocate the legalization or practice of prostitution or sex trafficking.” (section 7631(e)).

“No funds … may be used to provide assistance to any group or organization that does not have a policy explicitly opposing prostitution and sex trafficking […]” (section 7631(f)

Sex workers were thus offered rights-supporting, sex work-specific healthcare through this funding stream, but paradoxically, the organisations implementing these programmes had to oppose them and their livelihood strategies.

Despite ideologically denying sex worker agency and their material realities, this funding had widespread, positive consequences. The PEPFAR Programme reports that it has saved 26 million lives globally since its inception in 2003,^27^ and that it has been a key intervention in protecting sex worker individual health as well as public health. One of PEPFAR’s conceivably unintended consequences was to create opportunities for building solidarity and cohesion among sex workers.^28^ For example, the first African sex worker meeting was co-hosted by a foreign-aid supported health non-governmental organisation (NGO) in inner-city Johannesburg in 2009,^29^ while PEPFAR has supported responses to human rights-related barriers to HIV, tuberculosis (TB) services and gender, which in turn would bolster sex worker human rights defence and movement-building.

In early 2025, the Trump Administration stunningly issued executive orders that abruptly paused or terminated funding contracts for Key Populations health in Africa. This has devastating consequences in SA and further afield: crucial HIV and TB research projects—including clinical trials—were halted, various Key Population-specific clinics were closed, and thousands of nurses, social workers and counsellors were laid off.^30^ Various articles have described the far-ranging impacts,^31^,^33^ while a modelling study chillingly predicts that if all PEPFAR funding is lost and not replaced, up to 600 000 lives will be lost in SA alone in the next decade.^34^

At a time of crisis and uncertainty, some affected health NGOs initially instructed staff not to speak to the media or to post on social media about the funding cuts, thus further isolating anxious Key Populations and creating mistrust. One anonymous NGO staff member noted that this ‘silence felt and still feels like complicity and I’m battling shame about adhering to collective silence. Can we truly call ourselves humanitarians if we choose the safe, comfortable corner of silence over solidarity with the communities we serve?’^35^

In an effort to buffer the funding cuts and forge strategic advocacy approaches, a ‘Working Group for an Emergency Sex Work Plan’ was established in May 2025 that comprised sex workers, clinicians, allies and health and human rights NGOs. Members of the Working Group drafted key demands for the South African government that included the reinstatement of specialised Key Population health services and to expedite law processes to decriminalise sex work to mitigate violence and stigma. Working Group members were invited to endorse the demands, and thus derive a consensus Working Group advocacy position. However, despite the withdrawal of PEPFAR funds, some health NGO partners felt bound by the provisions in the PEPFAR Anti-prostitution pledge and/or were reticent to appear critical of the SA and US governments because of funding implications, and declined to sign on. With no consensus on key demands, the Working Group became largely defunct.

### How do sex workers define solidarity?

Insight into how sex workers define solidarity is useful here. In the sex work context, the term is generally associated with sex worker movement-building or with political changes to the legal position of sex work.^36 37^ The latter usually refers to sex workers and sex worker allies calling for sex work law reform in the form of decriminalisation and for safeguarding sex worker human rights.^38^ The Sisonke Sex Worker Movement in SA employs the slogan ‘Nothing about us without us’ to illustrate that no decisions about sex work should be taken without active consultation with sex workers. This is in resistance to paternalistic programmes that endeavour to ‘rescue’ sex workers or to ‘oppose’ them.

Thus, a precondition for solidarity with sex workers is to reject paternalism, to support meaningful participation of sex workers, and to recognise that sex workers and their livelihoods should not be treated as criminal.

### Forging new approaches: what would ‘sex worker solidarity’ look like?

Public health approaches to sex work have gradually shifted to recognise the importance of supporting sex worker human rights and to protect sex workers against HIV, violence and discrimination. For example, UNAIDS has supported this approach by popularising the principle ‘Leave no-one behind’ in its HIV response and the SDGs of ‘Ending AIDS’ by 2030 by ensuring the needs of the most underserved and stigmatised are met. More explicitly, in 2024 it noted its solidarity with sex workers—in a press release it stated ‘On International Sex Workers’ Day, 2 June 2024, and every day, UNAIDS stands in solidarity with sex workers in support of their health’.^39^

The case studies above have shown UNAIDS’s historically mottled approach to sex worker human rights and solidarity, which led eventually towards a commitment to principled partnership. Throughout this evolution, sex worker groups have had to advocate loudly for inclusion in international decision-making forums to ensure that health approaches to sex work and HIV were couched within human rights frameworks, within evidence, were as inclusive as possible and that barriers to meaningful participation were removed. Sex workers and their allies had to remain vigilant and ensure that gains would not be eroded.

### What would give meaningful content to solidarity?

Mtolo reminds us to be wary whenever solidarity is invoked, as it could serve as ‘the glue that holds people together in the absence of functioning systems, not a substitute for those systems themselves.’^40^ Calls for solidarity with sex workers—as articulated by sex workers—should therefore be linked to structural changes and action, not stop at homilies.

We see multiple opportunities for solidarity with sex workers at this time of ‘seismic global shifts’ in global health.^41^ The first step is to actively enquire from, and listen to, sex workers about their material realities and the problems they face, and to forge potential solutions in consultation with sex workers.^42^ Then to commit to move from the conceptual to the practical implementation of such solutions.

Resources are fundamental to implementation, and funds supporting sex worker health and human rights are haemorrhaging. National governments, international health and human rights agencies and philanthropies have to demonstrate political will and make the requisite financial resources available to continue—or re-start—evidence-based programmes to safeguard the health and rights of sex workers and other marginalised groups. Indeed, South African sex work researchers Milovanovica and Coetzee call financing of sex worker programmes a ‘moral imperative’.^43^ Against this background, the UNAIDS 2025 Global AIDS Update report references ‘solidarity’ 16 times (in contrast to the 2024 report that mentions solidarity only once, and not at all in 2023) and connects it mostly to ‘donor solidarity’—thus signifying how solidarity is irrevocably linked to money.

Solidarity with sex workers also requires courage and a principled commitment to speaking out against ideological-based funding restrictions and the far-reaching impact of US funding cuts. It is vital to be vocal about allyship and solidarity to Key Populations during times of crisis and uncertainty. Against this background, HIV researchers Hatcher and colleagues draw attention to the importance of resisting inward withdrawal and to consciously build community during the crisis brought about by the US funding cuts:

It can be tempting in this fast-moving and uncertain time to retreat. We may focus our attention on narrow actions that place our own research or practice at acute risk, rather than staying connected to and in solidarity with our colleagues and communities.^44^

Finally, when invoking solidarity with sex workers, it has to be bound to action that will support the transformation of social and structural drivers of sex worker ill health and inequality.^45^ This requires challenging the criminalisation of sex work, deeply unequal gender relationships, high levels of violence, sexual moralism and the criminalisation of poverty and informal livelihoods.

Employing solidarity as a lens through which to view global health dynamics could help to identify where the greatest vulnerabilities and dangers to collective health and well-being lie and to scrutinise underlying power structures and resources. We have shown how sex worker groups have had to insist on solidarity—or at least its proxies, recognition and partnership—from unwilling international global health bodies over many years.

Often solidarity is not freely given. From the case studies above, we have learnt that the content of solidarity should be carefully described and articulated by those who are calling for it, followed by explicit demands for these by collective action and persistence. While solidarity could remain just a ‘beautiful notion’ that is aspirational but potentially limp, it has more transformational promise when it functions as a political tool for social change.

## Data Availability

Data are available upon reasonable request.
